# Managing Rheumatoid Arthritis with Dietary Interventions

**DOI:** 10.3389/fnut.2017.00052

**Published:** 2017-11-08

**Authors:** Shweta Khanna, Kumar Sagar Jaiswal, Bhawna Gupta

**Affiliations:** ^1^Disease Biology Laboratory, School of Biotechnology, KIIT University, Bhubaneswar, Odisha, India

**Keywords:** rheumatoid arthritis, diets, foods, essential fatty acids, synbiotics

## Abstract

Self-help by means of dietary interventions can help in management of various disorders including rheumatoid arthritis (RA), a debilitating autoimmune disease. Dietary interventions necessitate a widespread appeal for both patients as well as clinicians due to factors including affordability, accessibility, and presence of scientific evidences that demonstrate substantial benefits in reducing disease symptoms such as pain, joint stiffness, swelling, tenderness and associated disability with disease progression. However, there is still an uncertainty among the community about the therapeutic benefits of dietary manipulations for RA. In the present review, we provide an account of different diets and their possible molecular mechanism of actions inducing observed therapeutic benefits for remission and management of RA. We further indicate food that can be a potential aggravating factor for the disease or may help in symptomatic relief. We thereafter summarize and thereby discuss various diets and food which help in reducing levels of inflammatory cytokines in RA patients that may play an effective role in management of RA following proper patient awareness. We thus would like to promote diet management as a tool that can both supplement and complement present treatment strategies for a better patient health and recovery.

## Introduction

Rheumatoid arthritis (RA) is a systemic, debilitating, chronic inflammatory autoimmune disorder affecting approximately 1% of the world population (1). The disease severely impacts quality of life with increased morbidity and reduced life expectancy. With the rapidly expanding population with RA, the disease has put a lot of economic burden on the society (2–4). Direct costs to governments are substantial while indirect costs owing to morbidity and mortality can be limiting for effective progress of a developing nation (2).

With undefined pathogenesis, different studies report a blend of environmental and genetic factors responsible for full expression of the disease. The shared epitopes coded by human leukocyte antigen (HLA) alleles, non-HLA genes, epigenetic factors, and differentially glycosylated proteins are considered significant risk factors for progression of RA (5–11). Elevated levels of rheumatoid factors (RF) (12), anti-cyclic citrullinated peptide autoantibodies (13) and anti-mannose-binding lectin autoantibodies (14) are some examples of autoimmune responses by RA patients. These factors, however, contribute approximately 50% to the risk of development of RA while the rest may be contributed by host–environment interactions (15). Environmental factors responsible for development of RA may present and act even before disease symptoms become apparent (13, 16). However, establishing the role of environmental factors in disease onset somehow becomes impossible due to concentration during disease onset (17). Early environmental factors such as high birth weight promotes chances of development of RA and early start of breast feeding reduces chances of development of RA (18). Other environmental factors such as smoking and infectious diseases also pose risk of developing RA (17).

The advances in understanding its pathogenesis have fostered the development of new and improved therapeutics; yet, with unknown cause and guarded prognosis, it is still an open field that requires special focus. The rate of progression is significantly rapid in the first few years of undetected or misdiagnosed RA (19). Early recognition and treatment of RA is complicated because of heterogeneous nature of the disease. No biomarker is available to detect the early onset of disease, and traditional biomarkers may not identify all patients that require early therapeutic interventions (20), and thus, the patients face severe complications with serious joint damage and disability. The first line of treatment for RA includes disease-modifying antirheumatic drugs (DMARDs) that suppress disease activity and reduce joint damages. With the development of better treatment strategies like biologic agents, e.g., anti-tumor necrosis factor (TNF)-α therapy (21) or combination of DMARDs with biologics, full remission could be achieved in a greater proportion of patients although a small group still show a frequent relapse post-discontinuation of TNF-α therapy (22). Continuous administration of biologics being the only option for prolonged remission, however, this being expensive (23) is still beyond the reach of most people in the urban and the rural sectors.

Furthermore, patients with RA generally complain of gastrointestinal tract problems particularly dyspepsia (bloating, postprandial fullness, nausea, early satiety, epigastric pain, and burning and belching), mucosal ulceration, and altered bowel habits (constipation/diarrhea) (24). An altered intestinal microbiota has thus been implicated in the etiopathogenesis of RA (25–27). Recently, Littman laboratory identified *Prevotella copri* significantly prevalent in RA patients than healthy controls providing the support that the “gut-joint axis” hypothesis is relevant for human rheumatic diseases and may lead to pathogenesis of RA (28). Rheumatologists do follow therapeutic regimens that target entero-arthropathy for rheumatic diseases, and several have been classified as DMARDs. Since 1940 sulfasalazine has been in use for the treatment of RA (29) and the triple DMARD therapy that combines hydroxychloroquine, sulfasalazine and methotrexate is still the first choice of treatment for most rheumatologists (30). As *Streptococci* found in milk was thought to be a cause of RA (31), sulfasalazine (combination of a sulfa antibiotic with a salicylate) has been proven efficacious for the treatment of RA (32). Proper mechanism of action of these drugs is not completely understood, despite the observation of encouraging clinical outcomes.

## Dietary Interventions in RA

**Figure 1:**
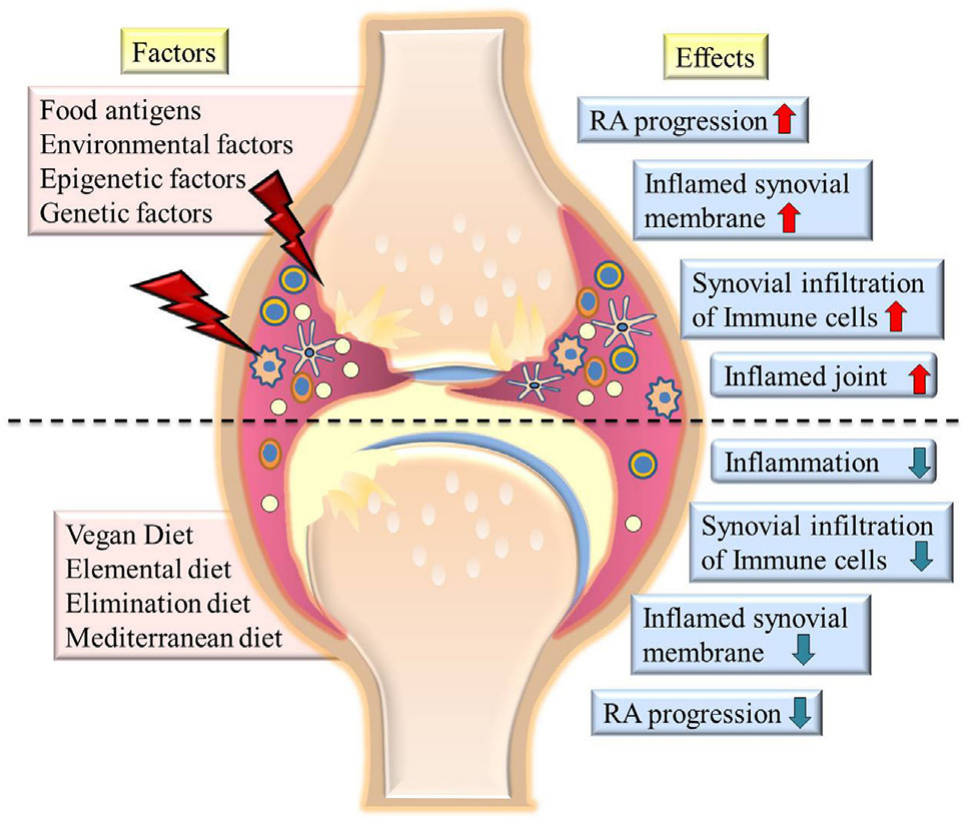
The picture summarizes various factors contributing to severity of rheumatoid arthritis (RA) and diets which cause remission of symptoms (left side of image). The effects of various factors on state of disease are shown at the right side of the image. The upper half of the image shows highly inflamed joints and synovial membrane, increased infiltration of immune cells in joints on exposure to environmental factors or food antigens. Lower half of the image shows effect of various diets in reducing inflammation, immune cell infiltration, and reducing the severity of disease.

With the increasing evidence of altered microbiota in the gut of RA patients being responsible for pathogenesis as well as disease progression (26, 28, 33), it should be desirable for rheumatologists to advocate a supplemental “diet therapy” to RA patients. Various dietary plans for RA have been reported since long (34) and are being repeatedly projected (35–39), such as medically supervised 7–10 days fasting (40–43), vegan (44–47) or Mediterranean diets (MDs) (48). We hereby discuss the reported dietary interventions that clearly indicate clinically and statistically significant and beneficial long-term effects for relieving symptoms, delay in disease progression and associated damages in RA patients. The outcomes of published randomized clinical trials performed on RA patients to observe the effect of various dietary interventions have been summarized in Table 1. A pictorial representation of effects put by various factors on progression/remission of RA is depicted in Figure 1.

**Table 1:** Summary of clinical trials of various dietary interventions in rheumatoid arthritis (RA).

Reference	Subjects, duration, and diet	Outcome
Kjeldsen-Kragh et al. (42)	*Diet group*—27 patients 7–10 days *subtotal fasting* (limited amount of nutritional supplements) 3.5 months on individually adjusted gluten-free vegan diet followed by lactovegetarian diet *Control group*—26 patients Ordinary diet throughout the study	After 1 month of diet Reduction in number of tender (*p* < 0.0002) and swollen joints (*p* < 0.04), Ritchie articular index (RAI) (*p* < 0.0004), pain (*p* < 0.0001), morning stiffness duration (*p* < 0.0002), grip strength, HAQ score, erythrocyte sedimentation rate (ESR) (*p* < 0.002), C-reactive protein (CRP) (*p* < 0.005), and WBC count (*p* < 0.0001) which were maintained even after 1 year of administration of diet *Key note*: Improvement can be maintained by continuing with individually adjusted diet
Kjeldsen-Kragh et al. (49)	*Diet group*—27 patients 7–10 days *subtotal fasting* 3.5 months on individually adjusted gluten-free vegan diet followed by lactovegetarian diet *Control group*—26 patients Ordinary diet throughout the study	After 1 month of treatment Significant decrease in leukocyte and platelet count (*p* < 0.003), IgM rheumatoid factors (*p* < 0.02), IgG, C3 (*p* < 0.04) and C4 complement components (*p* < 0.01), calprotectin (*p* < 0.03) and C3 activation products in diet responders in vegetarian diet group *Key note*: Dietary interventions can help in improvement of disease in some RA patients
Peltonen et al. (50)	*Diet group*—27 patients 7–10 days *subtotal fasting* 3.5 months on individually adjusted gluten-free vegan diet followed by 9 months lactovegetarian diet administration *Control group*—26 patients Ordinary diet throughout the study	Significant difference in fecal fatty acid profile at different times during the dietary intervention as compared to baseline in diet group was observed (*p* < 0.005). Fecal flora was significantly different between vegan diet (post 1 month treatment) and lactovegetarian diet period (*p* < 0.001). Significant difference in fecal flora was also observed between high improvement to low improvement groups (*p* < 0.001). This difference was also found at 1 month (vegan diet) and 13 months (lactovegetarian diet) *Key note*: Study finds association between disease activity and intestinal flora indicating impact of diet on disease progression
Haugen et al. (51)	*Diet group*—27 patients 7–10 days *subtotal fasting* 3.5 months on individually adjusted gluten-free vegan diet followed by lactovegetarian diet *Control group*—26 patients Ordinary diet throughout the study	Post 3.5 months of vegan diet Significant reduction in plasma fatty acid 20:3n-6 (*p* < 0.0001) and 20:4n-6 (*p* < 0.01) was observed which reversed to baseline concentration after lactovegetarian diet Significant reduction in 20:5n-3 post-vegan diet (*p* < 0.0001) and lactovegetarian diet (*p* < 0.01) No significant difference in fatty acid concentration between diet responders and non-responders after vegan or lactovegetarian *Key note*: Change in fatty acid profile could not explain disease improvement
Haugen et al. (47)	*Diet group*—17 patients 7–10 days *fasting* 3.5 months on gluten-free vegan diet followed by 9 months lactovegetarian diet administration *Control group—*17 patients Ordinary diet throughout the study	After 1 month Significant reduction in body mass index (BMI) and triceps skin fold thickness in diet group as compared with baseline (post 1 month) (*p* < 0.001) and controls (post study) (*p* = 0.04; *p* < 0.01) *Key note*: One year of dietary intervention had a minor impact on nutritional status of patients. No significant differences in other clinical variables studied were observed between the two groups
Kjeldsen-Kragh et al. (44)	Patients of above study were (42, 49) called for follow-up; 1 year post-trial. All responders and half non-responders were still on diet. Most of the patients eliminated those food which they thought aggravated their disease	Diet responders showed greatest change in clinical variables including HAQ (*p* < 0.04) and RAI (*p* < 0.02) from the baseline. Significant improvements were observed in all clinical variables including pain (*p* < 0.005), morning stiffness duration (*p* < 0.005), tender joint (*p* < 0.0003), RAI (*p* < 0.0001) and swollen joints (*p* < 0.05) except grip strength as compared to non-responders and controls *Key note*: Patients gained benefit from manipulation of diet which can be maintained for long term
Kjeldsen-Kragh et al. (52)	*Diet group—*26 RA patients 7–10 days *fasting* followed by 3.5 months of gluten-free vegetarian diet	Agalactosyl IgG antibodies reduced in RA patients and correlated significantly (*p* = 0.04) with clinical improvement post fasting which was not observed after administration of vegetarian diet *Key note*: IgG glycosylation may improve disease status during fasting
Fraser et al. (53)	*Diet group*—10 patients 7 days *subtotal fasting* 13 patients—ketogenic diet for 7 days All patients followed 2 weeks period of re-feeding on lactovegetarian diet	Post 7 days fasting Significant decrease in serum IL-6 levels in fasting group (*p* < 0.03) on seventh day as compared to baseline and after re-feeding. Improvement was observed in ESR, CRP, and tender joint counts post 7 days fasting *Key note*: Fasting improves disease activity in RA patients
Michalsen et al. (54)	16 RA patients and 35 fibromyalgia patients 21 patients—vegetarian Mediterranean diet (MD) 30 patients—intermittent modified 8 days fasting therapy	No difference in the fecal bacterial counts, concentration of secretory immunoglobulin or pH of the stool within or between the two diet groups. Post 2 weeks of study, fasting RA patients showed more clinical improvement as compared to non-fasting patients *Key note*: Clinical improvement is not related to intestinal flora
Abendroth et al. (55)	22 patients—medical fasting for 7 days 28 patient—*MD*	Both groups observed significant decrease in disease activity score (DAS) (*p* < 0.001). Significantly higher decrease in pain in fasting group on seventh day (*p* = 0.049). No significant difference was observed in total fatty acid profile, butyrate and propionate but acetate increased significantly (*p* = 0.044) in fasting group and decreased significantly in MD group. No significant correlation between diet induced changes in short chain fatty acids and disease activity changes was observed *Key note*: Change of intestinal microflora and relation with diet needs further studies
Sköldstam et al. (48)	Diet group—26 patients—*MD* *Control group*—25 patients	After 12 weeks of study, MD group showed significant reduction in DAS28 score (*p* < 0.001), decrease in HAQ (*p* = 0.020), and improvement in SF-36 health survey in two dimensions (*p* = 0.018). Out of 14 efficacy variables, 9 had shown improvement in diet group *Key note*: MD administration reduced disease activity in RA patients
Hafström et al. (45)	*Diet group*—38 patients—*gluten-free vegan diet* *Control group*—28 patients	Vegan group showed higher response rate and significant improvement in all variables except CRP. The diet responders have significant improvement in CRP (*p* < 0.05). Levels of IgG anti-gliadin (*p* = 0.0183) and anti-β-lactoglobulin (*p* = 0.0162) levels have significantly reduced from baselines in vegan diet groups. After 6 and 12 months, there was significant increase in Larsen score, number of erosions and joint count in both groups *Key note*: Diet change may reduce immunoreactivity to certain food antigens and some RA patients and may have certain clinical benefits
Peltonen et al. (56)	*Diet group*—*uncooked vegan diet rich in lactobacilli* *Control group*—normal omnivorous diet.	Diet group had significant change in fecal microflora from pre-test and post-test samples (*p* < 0.001) but not in control group. Significant difference was found on comparison of test group with control group at 1 month (*p* < 0.001). Significant difference in microflora was observed between low and high improvement index group after 1 month (*p* = 0.001) and after intervention (*p* = 0.029) but not in pre-test samples *Key note*: Fecal microflora changes with diet and helps in improvement of RA
McDougall et al. (46)	24 RA patients—very *low fat vegan diet*	Significant decrease in energy intake (*p* < 0.001), fats (*p* < 0.001) and proteins (*p* < 0.001) and significant increase in carbohydrate intake (*p* < 0.001) with decrease in weight. RA symptoms decreased including pain (*p* < 0.004), morning stiffness (*p* < 0.04), joint swelling (*p* < 0.02), and tenderness (*p* < 0.01) with increased joint mobility (*p* < 0.001) *Key note*: RA symptoms significantly decrease in moderate or severe RA patients on administration of very low fat vegan diet
Elkan et al. (57)	*Diet group*—38 patients—*gluten-free vegan diet* *Control group*—28 patients	After 12 months, vegan group showed decreased BMI, LDL, and weight. DAS28 (*p* = 0.002) and HAQ scores (*p* = 0.010) decreased significantly in at least 3 months when compared to baseline and CRP decreased (*p* = 0.008) at 12 months. In vegan group, at least in 3 months, total cholesterol (*p* < 0.001), LDL (*p* < 0.001) and LDL/HDL ratio (*p* < 0.001) significantly decreased but TGs and HDL did not change. OxLDL significantly decreased (*p* = 0.021) after 3 months in responders group. IgM anti-phosphorylcholine increased significantly trend wise and was significant at twelfth month (*p* = 0.057) *Key note*: Vegan diet (gluten free) is anti-inflammatory and atheroprotective
Sköldstam et al. (58)	*Study 1: Diet group*—14 patients—*lactovegetarian diet* *Control group*—10 patients *Study 2*: 13 patients—control period of 2 months 7 patients—control period of 5 months followed by *vegan diet* for following 4 months *Study 3: Diet group*—26 patients—*Cretan MD* *Control group*—25 patients	*Study 1*: At end of study, diet group reported reduction in pain with a significant weight loss (*p* < 0.001) but no change in disease outcome and no change in control subjects were observed *Study 2*: During vegan diet, all 20 patients were reported to have significant reduction in pain score, increased functional capacity, and significant weight loss (*p* < 0.001), which was not observed during the control period *Study 3*: 9 out of 14 disease outcome measures were improved with a significant loss in weight (*p* < 0.001) and decreased pain when compared to controls Statistically significant correlation was found between diet and three disease outcome variables including ΔAcute-Phase Response (*p* = 0.007), ΔPain Score (*p* = 0.005), and ΔPhysical Function (*p* = 0.002) *Key note*: Improvement of RA on administration of Vegan, Mediterranean, or lactovegetarian diet is not related to reduction of body weight
Ågren et al. (59)	*Diet group*—16 patients—*vegan diet* *Control group*—13 patients	Significant reduction (*p* < 0.001) of serum total, LDL cholesterol, and phospholipid concentrations were observed in vegan diet group. Sitosterol concentration increased and that of campesterol decreased giving a significant greater ratio of sitosterol: campestrol (*p* < 0.001) in vegan diet group when compared to control group *Key note*: Serum cholesterol, cholestanol, phospholipids, and lathosterol decrease in uncooked vegan diet
Hänninen et al. (60)	42 patients divided in two groups—*Uncooked vegan diet* for 3 months and omnivorous control groups	The RA symptoms reduced in diet group and reverted on restarting omnivorous diet. There was a significant negative correlation between degree of subjective adaptation system and decreased activity of RA (*p* = 0.003) *Key note*: Vegan diet rich in fibers, antioxidants, and lactobacilli improved RA in some patients
Vaghef-Mehrabany et al. (61)	*Diet group*—22 patients—10^8^ colony-forming unit (CFU) of *Lactobacillus casei 01* for 8 weeks 24 patients—placebo with maltodextrin for 8 weeks	Number of tender and swollen joints, serum hs-CRP levels, DAS, visual analog scale (VAS) score, tumor necrosis factor (TNF)-α, and IL-12 decreased significantly in probiotic group. Significant increase in IL-10 (*p* = 0.02), IL-10/IL-12 (*p* = 0.01), and IL-10/TNF-α (*p* = 0.03) was observed in the probiotic group *Key note*: Disease activity and inflammatory status improved in patients on *L. casei 01* supplementation
Vaghef-Mehrabany et al. (62)	*Diet group*—22 patients—10^8^ CFU of *L. casei 01* for 8 weeks 24 patients—placebo with maltodextrin for 8 weeks	No significant difference was observed within or between probiotic and placebo group in serum malondialdehyde, total antioxidant capacity, and catalase activity. Erythrocyte superoxide dismutase activity decreased significantly in probiotic group and glutathione peroxidase activity decreased in both groups. Difference between two groups was insignificant for both groups at the end of the study *Key note*: Probiotic supplementation does not have significant effect on oxidative status of RA patients
Hatakka et al. (63)	*Diet group*—8 patients—*L. rhamnosusGG* (*LGG)* (≥5 × 10^9^ CFU/capsule), twice a day for 12 months 13 patients—placebo group	Mean number of tender and swollen joints decreased in probiotic group. A 71% reduction in disease activity was observed in probiotic group and 30% in placebo group. Serum IL-1β increased in probiotic group and decreased in placebo group. At the end of the study, fecal recovery of LGG was increased from 25 to 86% in probiotic from baseline and decreased from 23 to 0% in placebo group *Key note*: More patients administered with LGG reported subjective well-being
Zamani et al. (64)	*Diet group*—30 patients—*L. acidophilus* (2 × 10^9^ CFU/g), *L. casei* (2 × 10^9^ CFU/g), and *Bifidobacterium bifidum* (2 × 10^9^ CFU/g) 30 patients—placebo group received capsule filled with cellulose	Probiotic group observed significant decrease in DAS28 score (*p* = 0.01), serum insulin levels (*p* = 0.03), HOMA-B (*p* = 0.03), serum hs-CRP concentrations (*p* < 0.001), LDL cholesterol (*p* = 0.07), and total cholesterol (*p* = 0.09) compared to placebo group. No significant effect was observed in tender and swollen joints, VAS pain, glucose homeostasis parameters, biomarkers of oxidative stress, and lipid profiles after probiotic administration *Key note*: Patients had significant benefit by incorporating probiotic supplements in diet
Vaghef-Mehrabany et al. (65)	Diet group—22 patients—10^8^ CFU of *L. casei 01* 24 patients—placebo group received similar capsules with maltodextrin	No significant difference within or between group for anthropometric and demographic parameters, physical activity was observed. Serum lipid did not change within any group significantly or in between the groups *Key note: L. casei* 01 could not improve serum lipid in patients
Alipour et al. (66)	*Diet group*—22 patients—10^8^ CFU of *L. casei 01* 24 patients—placebo group	Probiotic decreased serum high sensitivity CRP levels (*p* = 0.009), counts of swollen (*p* = 0.003) and tender joints (*p* = 0.03), DAS (*p* < 0.05), and global health score (*p* = 0.00). Global health score decreased significantly in placebo group as well. At the end of study, more patients in probiotic group showed moderate response to the supplementation according to EULAR criteria but all were non-responders in placebo group. The difference of IL-6, IL-12 (0.00), TNF-α (*p* = 0.002), and IL-10 (*p* = 0.007) cytokines between the two groups was statistically significant *Key note*: Probiotic can be an adjunct therapy for relieving symptoms
de los Angeles Pineda et al. (67)	*Diet group*—15 patients—*L. rhamnosus GR-1* and *L. reuteri RC-14* with 2 billion CFU viable bacteria 14 patients—placebo	Significant difference was observed in HAQ score (*p* = 0.02) in probiotic group when compared to baseline but not between groups. The pro-inflammatory cytokines including GM-CSF, IL-6, IL-1α, TNF-α, and IL-15 decreased but not significantly in the probiotic group. No difference was observed in cytokine levels and DAS *Key note*: Probiotics did not improve RA but functional improvements were reported
Mandel et al. (68)	*Diet group*—22 patients—*Bacillus coagulans GBI-30, 6086* (2 billion CFU) with green tea extract, methylsulfonylmethane, and vitamins and minerals (including vitamins A, B, C, D, E, folic acid, and selenium) 22 patients—placebo group received microcrystaline cellulose	Probiotic group showed statistically significant improvement in patient pain assessment score (*p* = 0.052) and pain scale (*p* = 0.046) as compared to baseline. Improvement was observed in patient global assessment, patient self-assessed disability, and reduction in total CRP but statistical difference was not found in physician global assessment or physician assessment of painful and swollen joints. Ability to walk 2 miles was marginally significant (*p* = 0.072) and ability to participate in daily activities was more in probiotic group *Key note*: Adjunctive therapy with probiotics serves effective for RA patients
Kavanagh et al. (69)	*Diet group*—24 patients—*elemental diet 028* (E028) (4 weeks) followed by food reintroduction where food unlikely to cause intolerance were introduced first followed by those which were known to cause intolerance one at a time. Food worsening RA was eliminated 23 patients—control groups were given E028 as a substitute to any drink along with normal diet	After 4 weeks of elemental diet, the diet group showed significant increase in grip strength (*p* = 0.008), decrease in RAI (*p* = 0.006), and loss of weight as compared to control diet group. CRP concentrations were different between the two groups but not significant. Statistically significant correlation was observed between loss of weight and grip strength at 1 week (*p* = 0.009) and 4 weeks (*p* = 0.027) in the diet group *Key note*: Elemental diet may improve some parameters in RA patients
Podas et al. (70)	*Diet group*—21 patients—*elemental diet E028* 9 patients—oral prednisolone 15 mg/day	All clinical parameters of RA including early morning stiffness, VAS, RAI, and HAQ improved significantly (*p* < 0.05) in both groups. Clinical parameters were improved by 20% in 72% patients in elemental diet group as compared to 78% in steroid group *Key note*: A 2 week treatment with elemental diet is as effective as 15 mg/day of prednisolone in improvement of clinical parameters. RA may start within the intestine due to reaction to various food antigens
Holst-Jensen et al. (71)	*Diet group*—15 patients—*commercial liquid diet (TU)*. TU contains hydrolyzed soy protein, triglycerides and carbohydrates, methionine, tryptophan, vitamins, and trace elements and is lactose free *Control group*—15 patients	4 weeks of treatment caused statistical significant improvements in pain (*p* = 0.02), HAQ score (*p* = 0.03) and reduction in BMI (*p* = 0.001). After the study, the number of swollen joints, ESR and General assessment of health, average during the last week lowered but not statistically significant. No difference was observed in the control group. Only one patient in the diet group achieved complete remission *Key note*: Peptide diet can improve some subjective and objective parameters of the disease. This diet may help those patients who have diet aggravated RA
Van de Laar and Van der Korst (72)	*Diet group*—45 patients—*allergen free diet* 49 patients—*allergen restricted with lactoproteins and yellow dyes* During first 4 weeks, patients followed their normal diets followed by 4 weeks of assigned diets and then administration of normal diet for 4 weeks	No significant difference could be found in clinical effects between the allergen free and allergen restricted diet. Only 9 out of 94 patients enrolled in the study showed favorable response but the disease relapsed after readministration of usual diets *Key note*: Some patients have food-aggravated RA, and they can be controlled by administering allergen-free food
Karatay et al. (73)	20 patients—positive skin prick test (SPT) to food extracts 20 patients—negative SPT All patients first *fasted to most common allergenic food* for 12 days. Food challenge was performed for PPG with allergenic food and for PNG with corn and rice for 12 days. Followed which allergenic foods were removed from respective groups	On food challenge in PPG, ESR (*p* < 0.05), CRP (*p* = 0.001), TNF-α (*p* < 0.01), and IL-1β (*p* < 0.05) increased and was also observed on re-elimination of food. In PNG, pain decreased significantly (*p* < 0.05) on food challenge. At end of re-elimination phase, differences were observed in between two groups in pain, duration of stiffness, number of tender and swollen joints, CRP levels, and RAI but not in HAQ and ESR levels. 72% patients in PPG group and 18% in PNG group suffered from disease aggravation on food challenge which continued in re-elimination phase *Key note*: Diet changes on individual level may change disease activity in patients

## Seven Days Fasting Followed by Vegan Diet

Fraser et al. (74) observed that subtotal fasting where patients were allowed to have limited amount of vitamin and mineral supplementation, carbohydrate, and energy in form of vegetable juice decreased CD4^+^ lymphocyte activation and numbers. Activation of CD4^+^ T cell and further differentiation to Th1 and Th17 lineages are shown to be responsible for progression of RA (75). Thus, decreased T cell activation owing to 7–10 day fasting suggests a transient immunosuppression, thereby suppressing RA (74). Michalsen et al. have also shown beneficial effect of fasting on clinical improvement in RA patients as compared to non-fasting group, although the results were independent of alterations in intestinal flora (54).

A fasting of 7–10 days with partial nutrient intake of vegetable broth, herbal teas, parsley, garlic, and decoction of potatoes; juice extracts from carrots, beets, and celery; and a controlled daily energy intake followed by 1 year of a vegan diet as compared to omnivorous diet was studied in different trials (42, 54). Together these studies observed remarkable decrease in swollen and tender joints, pain, erythrocyte sedimentation rate (ESR), and C-reactive protein (CRP).

### Vegan Diet

A diet including intake of only fruits and vegetables, eliminating any animal product or by-products is vegan diet. This has been repeatedly reported to be clinically beneficial for disease remission in RA patients (44–46, 57). Studies conclude that the improvements in disease activity might have been a result of reduction in immune-reactivity to certain food antigens in the gastrointestinal tract that were eliminated by changing the diet (45, 46). Furthermore, Hafström et al. (76) observed that during fasting there was decrease in duration of morning stiffness, ESR, articular index, concentrations of acute-phase reactants including orosomucoid, C3 and haptoglobin and an increase in hemoglobin. Moreover, the release of lysozyme by neutrophils was reduced in RA patients, the components of which are known to cause inflammation and destruction of joints. Leukotriene B4 (LTB4) is a pro-inflammatory mediator, involved in activation of neutrophils, eosinophils, and monocytes, production of pro-inflammatory cytokines, which further leads to tissue inflammation and neutrophil-mediated tissue damage (77). It was reported that the release of LTB4 from neutrophils was markedly reduced at completion of the fasting week (76).

Furthermore, it has been reported that during starvation, ketone bodies, including β-hydroxybutyrate (BHB), increase and serve as an alternate source of ATP in mammals (78). NLRP3 inflammasome regulates release of IL-18 and IL-1β (pro-inflammatory cytokines) in macrophages and gets activated on receiving damage-associated molecular patterns (79). Yun-Hee Youm et al. reported inhibition of activation of NLRP3 inflammasomes by BHB in response to various NLRP3 activators. BHB also reduced NLRP3-mediated release of IL-1β and IL-18 from human monocytes. Thus, the study concludes that starvation or ketogenic diets may play an anti-inflammatory role through inflammasome inhibition in BHB-mediated manner (79). This has been as well reported in the review by Tedeschi and Costenbader (80).

Müller et al. conducted a meta-analysis in order to find the effect of fasting followed by vegetarian diet in patients with RA. The study reports clinically and statistically significant beneficial long-term effect on RA patients, which may be used as a treatment for the disease (81).

Therefore, fasting followed by vegan diet or vegan diet alone can potentially reduce symptoms and disease activity in RA patients independent of changes in intestinal microflora. Improvements observed can be attributed to reduced exposure to potential antigens contributed by the omnivorous diet of RA patients.

### Mediterranean Diet

Mediterranean diet is rich in oleic acid, omega-3 fatty acids, unrefined carbohydrates, and phytochemicals (82). MD and particularly the Cretan MD involve high consumption of olive oil, cereals, fruits, vegetables, fish, and legumes; less red meat; and inclusion of moderate amount of red wine in diet. A study conducted by Sköldstam et al. concluded that on administration of Cretan MD to RA patients, inflammation was reduced, vitality and physical functions were improved (48). An important component of MD is olive oil which has antioxidant properties, is rich in oleic acid (18:1n-9), is metabolized to form eicosatrienoic acid (20:3n-9), and has anti-inflammatory effects similar to those of n-3 polyunsaturated fatty acids from fish oils (48). Studies have also shown that incorporation of olive oil in diet decreases the risk of developing RA (83). Rosillo et al. (84) have shown that administration of extra virgin olive oil in CIA mice (type II collagen-induced arthritis) reduced the serum levels of cartilage oligomeric matrix protein (COMP) and metalloproteinase-3 (MMP-3) that are the predictive markers of cartilage and joint damage in RA. The expression of pro-inflammatory cytokines including IL-1β, TNF-α, and IL-17, involved in progression of the disease, was also reduced. STAT-3 transcription factor promotes abnormal growth and prolonged survival of synovial cells (85) as well as in Th17 cell differentiation (86) in RA. Rosillo et al. also concluded that olive oil diet interfered with STAT-3 signaling by suppressing phosphorylation of STAT-3 and thus repressing IL-17 production (84). MAPKs induce pro-inflammatory gene expression, thereby promoting inflammatory processes (87). On investigating the effect of dietary olive oil on MAPKs (JNK and p38) signaling pathway in mice fed with olive oil, they found reduced levels of phosphorylated JNK and p38 proteins. They also observed reduced translocation of p65 to nucleus thus reducing NF-κβ mediated activation of various pro-inflammatory genes including TNF-α, IL-17, IL-6, and IL-1β within arthritic joint microenvironment where they can influence osteoclast differentiation thus promoting joint destruction. Therefore, reduction in NF-κβ mediated activation of pro-inflammatory cytokines will minimize joint destruction in patients. The study concluded that mice fed with olive oil had reduced cartilage destruction, joint edema, and arthritis development, and thus, olive oil may be beneficial in preventing RA (84).

### Elemental Diet

Elemental diet provides food in simplest form consisting of glucose, vitamins, trace elements, and essential amino acids, is hypoallergenic, contains all nutrients for daily requirements, and is thought to be less immunogenic (71). In the clinical trial conducted by Podas et al. (70), RA patients were given an elemental diet (E028) providing 86 kcal and 2.5 g protein/100 ml liquid elemental diet for 2 weeks. A large proportion of patients (72%) taking this elemental diet had more than 20% improvement in pain [on a 10 cm visual analog scale (VAS)], early morning stiffness, and the Ritchie articular index (RAI). The study concluded that this diet was as effective as 15 mg/day of oral prednisolone. However, no improvement was visible in the laboratory parameters including ESR, CRP, hemoglobin and a relapse of symptoms on discontinuation of this elemental diet pointed toward food antigens playing a possible role in pathogenesis and progression of RA (70). Similarly, Kavanagh et al. (69) and Holst-Jensen et al. (71) reported effects of different elemental diets with improvement in clinical and symptomatic parameters helping patients with food-aggravated disease conditions. Patients treated with elemental diet showed reduced symptoms of RA but relapsed on discontinuation (69). These studies further indicate that aggravation in symptoms of RA may be an effect of certain food allergens that are absent in elemental diet.

### Elimination Diet

Certain food and food components may worsen the disease conditions in RA (69, 70). Thus, an elimination diet plan may as well be considered wherein we eliminate those food related antigens that may possibly aggravate the disease symptoms (72). Intestinal epithelium is an interface between mucosal immune system and external environment, and it is the interaction between intestinal epithelial cells and mucosal immune system which determines the resultant immune response to various food antigens (88). There are many evidences that show food as a potential antigen for humans which pass through the gastrointestinal tract’s epithelium and further interact with mucosal immune system and move into circulation (89). It has been shown that the intestinal mucosa is more permeable to allergens in RA patients on administration of non-steroid anti-inflammatory drugs (90). A study conducted by Van de Laar and Van der Korst (72), included seropositive RA patients divided into two groups of which one was administered diet free of additives, allergens, and preservatives and other was on allergen restricted diet containing yellow dyes and lactoproteins. No difference was observed in clinical effects on RA patients taking any of these diets (72). A study conducted by Karatay et al. enrolled 18 RA patients who gave a positive skin prick test (SPT) (PPG) response to at least one food and 17 RA patients with completely negative SPT (PNG) results. All patients were kept on elimination diet where patients in PPG were given prick positive food and PNG patients were given corn (most allergenic to RA patients) along with rice (not allergenic) in increased amount for 12 days. This phase was then followed by re-elimination phase. In PPG, ESR, CRP, pain, tender and swollen joints, RAI score, TNF-α, and IL-1β increased during the challenge phase and after re-elimination phase. Thus, these studies concluded that food allergens are potential triggers of the immune system leading to inflammation by the activation of macrophage and other effector cells.

Treatment of RA includes inhibition of TNF-α and IL-1, and these inflammatory mediators are observed to be increased with the intake of allergenic food hence excluding some of these food from RA patient’s diet may benefit them as well as help them to reduce their requirement of recombinant human IL-1 receptor antagonist and anti-TNF-α antibodies (73).

## Individual Food Items in Diet and Their Relevance to RA

In an average diet comprising of breakfast, lunch, and dinner, there are several food items which are rich source of some phytochemicals and their efficacy in eradication of diseases has been known and is included under traditional medicines on which 80% of the world population relies (91). Food items such as dietary fibers, cooking oil, polyphenols, bioactive compounds from several herbs and beverages like tea are among the cheapest sources of medication; however, their bioavailability has always been a matter of concern.

### Dietary Fibers and Whole Grains

Most of the staple food consumed all over the world are comprised of dietary fibers and whole grains. A definitive explanation for dietary fibers can be put as remnants of food not digested in small intestine, which then moves to large intestine and gets fermented by the microflora and induces several health promoting effects (92). Insoluble fibers such as cellulose and lignin are found in fruits, vegetables, and whole grains; and soluble fibers include pectin, guar gum, and mucilage (93). Earlier studies have found an inverse relationship between intake of dietary fiber and inflammatory biomarkers such as plasma fibrinogen, hs-CRP, TNF-α, IL-6 levels which are indicators of RA (94). However, contradictory reports were published as well by Hu and the group (95).

When germ, endosperm, and bran are present in same proportions as in intact grains, they are regarded as whole grains. Whole wheat, whole rice, oats, corn, rye, barley, millets, sorghum, canary seed, fonio, and wild rice are generally included in the category of common whole grains (96). Whole grains provide rich amounts of antioxidants, phytic acid, vitamin E and selenium, and these components are known to be involved in anti-inflammatory processes (97).

Even if no conclusive evidences are found about the role of dietary fibers and whole grains in RA, Food and Drug Administration (FDA) has approved their health promoting claims (98). As per Dietary Reference Intakes recommendations, daily consumption of dietary fibers within the limit of 14 g per 1,000 kcal intake or 25 and 38 g for an adult women and men, respectively (93) has health benefits.

### Fruits

Apart from the botanic definition, fruits are the pulpy seeded tissues with sweet and tart taste (99). Bioactive components and phytochemicals, the non-nutrient plant compounds, present in fruits and vegetables are the key players and have been shown to diminish the symptoms of several chronic diseases such as atherosclerosis, arthritis, diabetes, asthma, AIDS, neoplasia, and cardiovascular diseases (100–102). Dietary phytochemicals are generally categorized into main groups as nitrogen-containing compounds, phenolics, organosulfur compounds, alkaloids, phytosterols, and carotenoids (103).

Regular consumption of fresh fruits rich in important phytochemicals can reduce oxidative stress and inflammation (104). Several cohort studies have also reported that repeated and high consumption is not only associated with downregulation of disease progression but also may provide protective effects against RA (105–107).

In patients suffering from RA, osteoclastogenesis (the process of bone tissue destruction by osteoclast cells) has been identified as a clinical phenomenon (108). Dried plums are rich source of polyphenols, when consumed can suppress osteoclastogenesis by inhibiting the activity of TNF-α and nitric oxide (NO) synthase and downregulate the transcription factor-nuclear factor for activated T cells (NFATc1) (109).

Anthocyanins have proved themselves as potent antioxidants and are more abundant in black rice, eggplant, and black soybean. These have properties to reduce oxidative stress by increasing superoxide dismutase (SOD) and decreasing serum malondialdehyde (MDA). It has been reported in mouse models of RA that the uptake of anthocyanins can bring down TNF-α levels (110), thereby reducing disease activity. Resveratrol from black grapes has been found to exert protective effect in rat model of RA (111). It was reported that resveratrol can lower down specific RA biomarkers such as serum RF, COMP, and MMP-3; immunological biomarkers as IgG and antinuclear antibody; immunomodulatroy cytokines (TNF-α) and oxidative stress (111). Mangiferin, a polyphenolic compound found in mangoes, used in an *in vivo* study on RA-induced DBA-1/J male mice reported downregulation of IL-1β, IL-6, and TNF-α, inhibited NF-κβ signaling, and activated extracellular signal regulated kinase 1/2 (ERK1/2) (112). In another study with mangiferin, it was observed that mangiferin prevented joint destruction in RA by inducing proapoptotic effects on human synovium-derived synoviocytes (113).

Kaempferol, an important phytochemical found in grapefruits, can bring down the level of inflammatory cytokine IL-1β, inhibiting the cell signaling pathways like phosphorylation of ERK1/2, p38, and JNK and activation of NF-κβ (114). Several enzymes inducing oxidative stress such as MMPs, COX-2, and PGE-2 in RA-derived synoviocytes were lowered down on administration of kaempferol (114). These molecules are reported in destruction of bone and articular cartilage leading to pathogenesis of RA (115, 116). A mixture of polyphenols composed of epigallocatechin, gallate, catechin, tannic acid, and querectin when injected at intra-articular region of rat model of RA, prevented cartilage destruction while reducing inflammation (117).

p-Coumaric acid is largely present in grapes, oranges, apples, tomatoes, spinach, and potatoes. In an *in vivo* study using rat model of adjuvant-induced arthritis, p-coumaric acid intake significantly reduced the expression of TNF-α (118). Genistein, an important isoflavone present in soybeans maintained a perfect balance between T helper cell, Th1 and Th2, and inhibited IFN-γ and IL-4 production which ultimately brings down the inflammation (119). Freshly prepared orange juice has high content of beta-cryptoxanthin and its intake reduces the risk of RA in humans (120). Pineapple stem are rich source of proteolytic enzyme called as bromelain. In a study, bromelain was consumed orally by RA patients in dosages of 20 or 40 mg for 3–4 times daily up to 13 months. About 72% of the total patients involved in the study came up with promising results, and there were no side effects detected. In spite of promising results obtained, significance of the study cannot be explained due to lack of control groups (121).

### Spices

Ginger has been known for its therapeutic properties due to the presence of pungent phenolics such as shogaols and gingerols (122). Turmeric, rich in phenolic curcuminoids, has also proved its beneficial effects against several malignancies (123). In a study, a perfect mixture of blended ginger and turmeric were given to the adjuvant-induced arthritic rats. This mixture showed protective effects against extra-articular complications of RA (122). In another study conducted by the same group, they found that ginger and turmeric administered at a dose of 200 mg/kg body weight could independently lower down the signs and symptoms of RA in the adjuvant-induced arthritic male Wistar albino rats. The results were significant with a *p*-value <0.05 as compared to the control group receiving only indomethacin (123).

Curcumin has also presented itself as a potent anti-inflammatory spice by blocking the expression of IL-1 and IL-6 in an *in vitro* study with RA patient-derived fibroblast-like synoviocytes (124). Methotrexate is a widely prescribed antirheumatic drug for the treatment of RA but it increases oxidative stress, decreases NO levels, and leads to vascular endothelial dysfunction (124, 125). Curcumin and folic acid co-administration was found to lower down methotrexate-induced vascular endothelial dysfunctions in male Wistar rats (126).

Bark of *Cinnamomum zeylanicum* (Cinnamon bark) is widely used in South-East Asian dishes. Rathi et al. treated RA animal models involving male Swiss albino mice and Wistar rats with polyphenolic fraction of cinnamon barks and found inhibitory effects on secretion of cytokines IL-2, IL-4, and IFN-γ and reduction in levels of TNF-α (127).

### Essential Fatty Acids

Omega-3 or omega-6 fatty acids have shown their potential as immunosuppressants and anti-inflammatory agents (128–131). Borage seed oil provides high amount of omega-6 fatty acid or gamma-linolenic acid (GLA) (132). A double-blind trial was conducted on 37 patients with active RA, and they were assigned to consume borage seed oil containing 1.4 g of GLA per day while placebo group was given cottonseed oil. After 24 weeks of consumption, the group which received GLA had significantly reduced tender and swollen joint scores, whereas placebo group did not show any change (133).

Gamma-linolenic acid and omega-3 fatty acid alpha-linolenic and stearidonic acid from black currant seed oil (BCSO) has also been investigated for their therapeutic activity. About 10.5 g of BCSO were given to RA patients in double-blind fashion and soybean oil as placebo for 24 weeks continuously. BCSO treated group, when compared with placebo group came up with significant positive effects in pain relieving and joint tenderness (134).

Fish oils provide high amount of omega-3 fatty acids, and their efficacy to treat RA has been checked in several controlled trials. RA patients were provided with fish oil with 3.6 g of omega-3 fatty acids per day in double-blind fashion, and placebo group were treated with mixture of fatty acids for 12 weeks, which was very much similar in amount found in average diet. The group which received fish oil had reduced morning stiffness, significant increase in grip strength compared to the placebo group (135). Eicosapentaenoic and docosahexaenoic acids are ethyl ester derivatives of omega-3 fatty acids, and their capability to reduce severity of RA has been assessed. When RA patients consumed these derivatives in an amount of 130 mg/kg body weight/day for 26–30 weeks, a significant decrease in pain, morning stiffness, and tender joints was observed in comparison with the placebo group that received only corn oil (136).

### Synbiotics

Synbiotics are composed of probiotics and prebiotics (the non-digestible food products beneficial for growth of helpful bacteria in large intestine and provides health promoting effects) (137). Several reports have confirmed the reduction of oxidative stress in human body by consumption of synbiotics (138–141). As per FDA, probiotics are “live microorganisms which, when administered in adequate amounts, confer a health benefit on the host” (142). *Bifidobacterium* and *Lactobacillus* are the key strains widely used as probiotics in commercial, pharmaceutical, and nutraceutical products (143, 144). Many reports have frequently stated that the population of gut microbes gets altered in a person affected with RA (56, 145–147), and several animal studies have already proved that any alteration in gut microbiota corresponds to initiation of RA (148).

In several animal and human studies, the health promoting benefits of probiotics has been extensively assessed. When RA-induced animal models were fed *Lactobacillus casei*, it led to improvised health conditions by reduction in levels of pro-inflammatory cytokines such as IL-1β, IL-2, IL-6, IL-12, and IL-17, IFN-γ, and TNF-α, while upregulating the secretion of regulatory cytokines like IL-10 and TGF-β (149–152).

When yogurts fermented with live or heat killed *Lactobacillus rhamnosus* GG (LGG) and *L. bulgaricus* were fed to arthritis induced Lewis rats, it significantly reduced arthritis clinical scores (153). Anti-inflammatory effects of methotrexate was enhanced, when the medicine combined with *Escherichia coli* strain O83 (Colinfant) was administered on adjuvant-induced arthritis models (154).

Different strains of probiotics have also been used for human studies with reports of health conditions improvements (63, 67, 68). Oxidative stress generated during metabolism has also been held as culprit for pathogenesis of RA and selective strains with high antioxidant activity may be employed to lower down disease progression. In a study, female RA patients were given *L. casei* 01 supplement capsules containing about 10^8^ colony-forming unit (CFU)/capsule and the placebo group maltodextrin for 8 weeks. After treatment, a significant decrease was observed in number of tender or swollen joints, VAS scores, hs-CRP levels, disease activity score (DAS), TNF-α, and IL-12 in the probiotic group with a significant increase in serum IL-10 levels. Alteration of gut microbiota is known in case of early RA disease, and probiotics normalize the gut fauna toward a normal healthy microbiota and show anti-inflammatory activity. At the end of the study, several oxidative stress indices were also measured wherein MDA level decreased insignificantly, total antioxidant capacity levels and catalase activity increased in probiotics groups while there were no changes observed in SOD and glutathione peroxidase activity (62).

In a pilot study conducted on 21 RA patients, the effect of LGG on their health condition has been assessed. Patients from test group were prescribed to take two capsules of LGG twice a day (Gefilus, Valio Ltd.; ≥5 × 10^9^ CFU/capsule), and the placebo group took the same capsule without bacteria for 12 months and finally several inflammatory parameters were measured. The number of tender and swollen joints reduced from 8.3 to 4.4, as compared to an increase from 5.5 to 5.6 in placebo group, mean serum IL-1β decreased in placebo group but no significant change in levels of TNF-α, IL-6, IL-10, IL-12, and myeloperoxiedase was observed (63).

### Alcohol Consumption

Consumption of alcohol with pathogenesis of RA is still under debate. While some studies point that alcohol consumption leads to progression of RA (155–158), others have concluded that no such relationship exists (159, 160).

In a recent case–control study on Scandinavian population, alcohol consumption led to decrease in RA risk in a dose-dependant manner when alcohol consuming subjects were compared with non-drinkers despite of their gender, age, and CCP status difference (161).

Another study focused on frequency of alcohol consumption not the amount, by RA patients of Caucasian ethnicity and reported similar results. All measures of RA severity such as CRP, DAS28 score, modified health assessment questionnaire, and pain VAS were found to be in inverse relation with increased frequency of alcohol uptake (80, 162).

### Tea

Epigallocatechin-3-gallate (EGCG) has proved its therapeutic potential and has been of particular interest among natural products for its use as a nutraceutical (163). It is a main phytochemical present in green tea that is obtained from dried leaves of *Camellia sinensis* and *C. assamica* of Theacease family (164). The protective effects exerted by green tea have been well proved in neurodegenerative disease, inflammatory disease, cardiovascular disease, and several types of cancer (165, 166).

In RA, the resistance of synovial fibroblasts against apoptosis has been set as a trademark, and this characteristic is enhanced by constitutive expression of proteins like AKT and NF-κβ and overexpression of Mcl-1 and Bcl-2 (anti-apoptotic proteins) (167). EGCG treatment has successfully shown its ability to downregulate Mcl-1 in synovial fibroblasts and increases the susceptibility toward apoptosis (167). The reports also conclude that EGCG successfully suppresses the production of MMP-1, MMP-2, and MMP-3 in synovial fibroblasts and prevents bone and cartilage destruction (168, 169). EGCG treatment in RA patients inhibits IL-1β induced IL-6 production by synovial fibroblasts and can upregulate an inhibitor, i.e., soluble gp130 receptor, which in turn suppresses IL-6 trans signaling (170).

### Herbs

Plants with effective health promoting effects are known as herbs, and these have a long history of being used as medicine to cure several diseases. Synthetic drugs used in arthropathies have been associated with numerous side effects on health, which in return has led the focus toward medicines of botanical origin (171).

Sallaki (*Boswellia serrata*) is widely recommended as an anti-inflammatory herb as prescribed in Ayurveda (172). The phytochemical which act as key player is boswellic acid from pentacyclic triterpene family (173). Boswellic acid inhibits the expression of lipoxygenase-5 and eventually lowering down leukotriene synthesis and leukotreines are well known for their role in inflammation (174–176). These have also proved their potency to block NF-κβ activation and brought down the levels of pro-inflammatory cytokines like TNF-α, IL-1, IL-2, IL-4, IL-6, and IFN-γ and also prevented classical complement pathway by restricting the cleavage of C3 to C3b (177).

Ashwagandha (*Withania somnifera*) is one of the plants being described in Ayurveda as a potent anti-inflammatory plant (178). It is rich in Withaferin A, a steroidal phytochemical which can prevent proceeding of NF-κβ signaling pathway (179). *In vitro* studies with ashwagandha extract suppressed release of pro-inflammatory cytokines as TNF-α, IL-12, and IL-1β from synoviocytes of RA patients but it failed to stop synthesis and subsequent release of IL-6 (180). Rats with induced arthritis when treated with powder of ashwagandha roots showed less destruction of bone collagen (181). Moreover, in a double-blind placebo-controlled study aqueous extract significantly reduced stiffness, disability to move knee and joints, and pain score (182).

## Conclusion

With the growing wealth of literature supporting the positive impact of diet therapy in decreasing disease activity in RA, with increasing understanding of microbiota mediated disease pathology and the beneficial effects of nutrients on inflammation and immunity, our interest in dietary interventions is growing. Patients are always interested in alternative treatments to relieve their debilitating condition. We believe that one should promulgate diet therapy for RA patients. Besides the regular DMARDs and anti-TNFs that are provided for effective cure of severe RA, patients should be motivated to change their eating habits. We should work to educate and capacitate them with the benefits of eating more vegetarian/vegan diets, eliminate potentially allergic food components, and introduce more poly unsaturated fatty acid/oleic acid/synbiotics in their diet plans. Early signs of RA can be potentially delayed with these dietary interventions. Considering that these food are not as expensive as any regular therapeutics, they can be easily incorporated for patients from any societal or economical background. Although it will be difficult to observe immediate benefits of these dietary manipulations, the long-term benefits are already reported.

We believe that an ideal meal can include raw or moderately cooked vegetables (lots of greens, legumes), with addition of spices like turmeric and ginger (123), seasonal fruits (183), probiotic yogurt (184); all of which are good sources of natural antioxidants and deliver anti-inflammatory effects. The patient should avoid any processed food, high salt (185), oils, butter, sugar, and animal products (186). Dietary supplements like vitamin D (187, 188), cod liver oil (189, 190), and multivitamins (191) can also help in managing RA. This diet therapy with low impact aerobic exercises can be used for a better degree of self-management of RA with minimal financial burden (192–194). A better patient compliance is, however, always necessary for effective care and management of RA.

Based on findings discussed in this review, we have designed an anti-inflammatory food chart (Table 2) that may aid in reducing signs and symptoms of RA. This may not cure the patients; however, an effective incorporation of these food items in the daily food plan may help to reduce their disease activity, delay disease progression, and reduce joint damage, and eventually a decreased dose of drugs administered for therapeutic treatment of patients.

**Table 2:** Recommended anti-inflammatory food chart.

Fruits	Dried plums, grapefruits, grapes, blueberries, pomegranate, mango (seasonal fruit), banana, peaches, apples
Cereals	Whole oatmeal, whole wheat bread, whole flattened rice
Legumes	Black soybean, black gram
Whole grains	Wheat, rice, oats, corn, rye, barley, millets, sorghum, canary seed
Spices	Ginger, turmeric
Herbs	Sallaki, ashwagandha
Oils	Olive oil, fish oil, borage seed oil (in encapsulated form)
Miscellaneous	Yogurt (curd), green tea, basil (tulsi) tea

## Author Contributions

SK, KJ, and BG designed the concept and were involved in writing of the manuscript.

## Conflict of Interest Statement

The authors declare that the research was conducted in the absence of any commercial or financial relationships that could be construed as a potential conflict of interest.
